# Methylation‐Specific Droplet Digital PCR: Testing a Novel Triage Tool for HrHPV‐Positive Women in the Cervical Cancer Screening Program of Northern Portugal

**DOI:** 10.1002/mco2.70203

**Published:** 2025-06-15

**Authors:** Sofia Salta, José Pedro Sequeira, Bárbara Amorim‐Fernandes, João Lobo, Inês Baldaque, Paula Monteiro, Fernando Tavares, Valérie Taly, Rui Henrique, Carmen Jerónimo

**Affiliations:** ^1^ Cancer Biology & Epigenetics Group Research Center of IPO Porto (CI‐IPOP) Portuguese Oncology Institute of Porto (IPO Porto) Porto Comprehensive Cancer Center Raquel Seruca (Porto.CCC) R. Dr. António Bernardino de Almeida Porto Portugal; ^2^ Doctoral Program in Molecular Pathology and Genetics ICBAS–School of Medicine and Biomedical Sciences–University of Porto Rua Jorge Viterbo Ferreira Porto Portugal; ^3^ Doctoral Program in Biomedical Sciences ICBAS–School of Medicine and Biomedical Sciences–University of Porto Rua Jorge Viterbo Ferreira Porto Portugal; ^4^ Department of Pathology Portuguese Oncology Institute of Porto (IPO Porto) Porto Comprehensive Cancer Center Raquel Seruca (Porto.CCC) R. Dr. António Bernardino de Almeida Porto Portugal; ^5^ Department of Pathology and Molecular Immunology ICBAS–School of Medicine and Biomedical Sciences–University of Porto Rua Jorge Viterbo Ferreira Porto Portugal; ^6^ Department of Pathology and Laboratory Medicine Clinical Pathology Service Portuguese Oncology Institute of Porto (IPO Porto) Porto Comprehensive Cancer Center Raquel Seruca (Porto.CCC) R. Dr. António Bernardino de Almeida Porto Portugal; ^7^ Departamento de Estudos e Planeamento Coordenação Nacional dos Programas de Rastreio de Base Populacional (CN‐Rast_Pop) Direção Executiva do Serviço Nacional de Saúde Alameda Professor Hernâni Monteiro Portaria B Porto Portugal; ^8^ Centre de recherche des cordeliers INSERM Sorbonne Université Université de Paris Cité Équipe 26 Équipe labellisée ligue nationale contre le cancer Paris France

**Keywords:** cervical cancer screening, colposcopy referral, DNA methylation, HPV‐positive women referral, triage

1

Dear Editor,

Cervical cancer remains a major global health problem. The most recent European guidelines endorsed HrHPV testing as the most cost‐effective primary method for cervical cancer screening, with cytology being proposed as a triage test [1]. Although highly sensitive, this strategy depicts limited positive predictive value (PPV) [1], entailing increased referral to colposcopy units [1], ultimately leading to overdiagnosis and overtreatment. Indeed, we found that, in the Northern Region of Portugal, using cytology combined with HPV16/18 genotyping as triage for HrHPV‐positive women, a fraction of those sent to the cervical pathology unit did not benefit from it [2]. Thus, alternative triage methods, such as dual p16/Ki67 staining (also known as CINtec PLUS) and DNA methylation markers, have emerged [3, 4]. Previously, we investigated the potential of methylation biomarkers for cervical cancer screening in the Portuguese population [5] and found that *MAL, FAM19A4*, and *hsa‐miR124‐2* promoter methylation levels were significantly higher in high‐grade intraepithelial (HSIL) or worse (HSIL+) lesions in both compared tissue and cervical scrape samples [5]. However, some technical limitations became apparent, including the need for preamplification steps for the analysis of cervical scrapes [5]. More sensitive technologies for methylation assessment, such as droplet digital PCR (ddPCR), are required to overcome this limitation. Thus, we sought to develop a droplet digital methylation‐specific PCR (ddMSP)‐based assay to be used as a triage tool for referral to colposcopy among HrHPV‐positive women in a well‐established population‐based cervical cancer screening program.

For this study, two patient cohorts (exploratory and replication) were selected. Relevant clinical data is depicted in Table S1. Leftovers from cervical scrapes primarily used for routine testing of women enrolled in the Regional Cervical Cancer Screening Program of Northern Portugal (RCCU‐NP) were selected for this project. Further information may be found in Supporting Information.

For the exploratory series, 62 samples were selected from a previously characterized cohort of patients who tested positive for HrHPV and were referred to colposcopy between March and May 2019 [5]. For the replication series, all samples received in January 2020 that tested positive for HrHPV were considered eligible, except those without clinical information or enough DNA stored, which were excluded from analysis. DNA from all cervical scrapes collected was bisulfite treated and used for ddMSP assessment of *MAL* and *hsa‐miR124‐2* methylation levels (see protocols’ details in Supporting Information). Biomarker performance was assessed and compared to the current strategy applied in the RCCU‐NP.

For the exploratory cohort, *MAL* and *hsa‐miR124‐2* promoter methylation levels significantly differed between HSIL+ and Normal & LSIL groups (*p* = 0.0013 and *p* = 0.0344, respectively). Additionally, 88.9% (CI 95%: 70.2–98.1) and 91.1% (CI 95%: 82.8–99.4) specificity for *hsa‐miR124‐2^me^* and *MAL^me^* was achieved, respectively (Table 1A). Combining both markers, 80.0% specificity was disclosed, although with limited sensitivity (47.1%), reaching 70.0% accuracy.

**TABLE 1:** Biomarker performance of methylation and HPV genotyping for detecting high‐grade intraepithelial lesions or worse in the exploratory and replication series, for proposed and current strategies of triage for HrHPV‐positive women.

A—Exploratory series performance for HSIL+ detection							
Biomarker	Normal & LSIL, *n* = 45, *n* (%)^a^	HSIL+, *n* = 17, *n* (%)^a^	Specificity %, (CI 95%)	Sensitivity %, (CI 95%)	PPV %, (CI 95%)	NPV %, (CI 95%)	Accuracy, (CI 95%)
*hsa‐miR124‐2^me^*	5 (11.11)	5 (29.41)	88.89 (79.71–98.07)	29.41 (7.75–51.07)	50.00 (19.01–80.99)	76.92 (64.61–89.23)	72.58 (61.48–83.69)
*MAL^me^*	4 (8.89)	7 (41.18)	91.11 (82.80–99.43)	41.18 (17.78–64.57)	63.64 (35.21–92.06)	80.39 (68.79–91.99)	77.42 (67.01–87.83)
*hsa‐miR124‐2^me^/MAL^me^*	9 (20.00)	8 (47.06)	80.00 (68.31–91.69)	47.06 (23.33–70.79)	47.06 (23.33–70.79)	80.00 (68.31–91.69)	70.97 (59.67–82.27)

B—Replication series performance for HSIL+ detection									
Triage algorithms	Normal & LSIL, *n* = 658, *n* (%)^a^	HSIL+, *n* = 92, n (%)^a^	Specificity %, (CI 95%)	Sensitivity %, (CI 95%)	PPV %, (CI 95%)	NPV %, (CI 95%)	Accuracy, (CI 95%)	FPR, (CI 95%)	Referal Rate (%)
*hsa‐miR124‐2^me^*	43 (6.53)	16 (17.39)	93.47 (91.58–95.35)	17.39 (9.65–25.14)	27.12 (15.77–38.46)	89.00 (86.61–91.39)	84.13 (81.52–86.75)	6.53 (4.65–8.42)	7.9
*MAL^me^*	35 (5.32)	17 (18.48)	94.68 (92.97–96.40)	18.48 (10.55–26.41)	32.69 (19.94–45.44)	89.26 (86.89–91.62)	85.33 (82.80–87.87)	5.32 (3.60– 70.3)	6.9
*hsa‐miR124‐2^me^/MAL^me^*	71 (10.79)	23 (25.00)	89.21 (86.84–91.58)	25.00 (16.15–33.85)	24.47 (15.78–33.16)	89.48 (87.14–91.83)	81.33 (78.54–84.12)	10.79 (8.42–13.16)	12.5
*hsa‐miR124‐2^me^/MAL^me^* + HPV16/18/33	151 (22.94)	64 (69.57)	77.05 (73.84–80.26)	69.57 (60.16–78.97)	29.77 (23.66–35.88)	94.77 (93.06–96.47)	76.13 (73.08–79.18)	22.95 (19.74–26.16)	28.7
*hsa‐miR124‐2^me^/MAL^me^* + HPV16/33	135 (20.52)	63 (68.48)	79.48 (76.40–82.57)	68.48 (58.98–77.97)	31.82 (25.33–38.31)	94.75 (93.04–96.45)	78.13 (75.18–81.09)	20.52 (17.43–23.60)	26.4
HPV 16/18 + Cytology (current)	226 (34.34)	72 (78.26)	65.65 (62.03–69.28)	78.26 (69.83–86.69)	24.16 (19.30–29.02)	95.58 (94.00–97.15)	67.20 (63.84–70.56)	34.35 (30.72–37.97)	39.7

C—Replication series performance for CIN3+ detection^b^									
Triage algorithms	Normal & CIN1/2, *n* = 694, *n* (%)^a^	CIN3 +, *n* = 41, *n* (%)^a^	Specificity %, (CI 95%)	Sensitivity %, (CI 95%)	PPV %, (CI 95%)	NPV %, (CI 95%)	Accuracy (CI 95%)	FPR (CI 95%)	Referal Rate (%)
*hsa‐miR124‐2^me^*	45 (6.48)	10 (24.39)	93.52 (91.68–95.35)	24.39 (11.25–37.54)	18.18 (7.99–28.38)	95.44 (93.89–96.99)	89.66 (87.46–91.86)	6.48 (4.65–8.32)	7.48
*MAL^me^*	41 (5.91)	6 (14.63)	94.09 (92.34–95.85)	14.63 (3.82–– 25.45)	12.77 (3.23–22.31))	94.91 (93.28–96.55)	89.66 (87.46–91.86)	5.91 (4.15–7.66)	6.39
*hsa‐miR124‐2^me^ /MAL^me^*	77 (11.10)	12 (29.27)	88.90 (86.57–91.24)	29.27 (15.34–43.20)	13.48 (6.39–20.58)	95.51 (93.97–97.05)	85.58 (83.04–88.12)	11.10 (8.76–13.43)	12.11
*hsa‐miR124‐2^me^ /MAL^me^* + HPV16/18/33	175 (25.22)	30 (73.17)	74.78 (71.55–78.01)	73.71 (59.61–86.73)	14.63 (9.80–19.47)	97.92 (96.86–98.99)	74.69 (71.55–77.84)	25.22 (21.99–28.45)	27.89
*hsa‐miR124‐2^me^ /MAL^me^* + HPV16/33	158 (53.74)	30 (73.17)	77.23 (74.11–80.35)	73.71 (59.61–86.73	15.96 (10.72–21.19)	97.99 (96.94–99.03)	77.01 (73.96–80.05)	22.77 (19.65–25.89)	25.58
HPV 16/18 + Cytology (current)	254 (36.60)	32 (78.05)	63.40 (59.82–66.98)	78.05 (65.38–90.72)	11.19 (7.54–14.84)	98.00 (96.95–99.04)	64.22 (60.75–67.68)	36.60 (33.02–40.18)	38.91

Abbreviations: CI, Confidence interval; CIN, Cervical Intraepithelial Neoplasia; CIN3+, Cervical Intraepithelial Neoplasia Grade 3 lesions or worse; FPR, False Positive Rate; HPV, Human Papillomavirus; HSIL+, High‐grade squamous intraepithelial lesions or worse; LSIL, Low‐grade squamous intraepithelial lesions; *n*, number; NPV, Negative Predictive Value; PPV, Positive Predictive Value.

^a^ Number of positive cases and percentage of the total.

^b^ Cases classified only as HSIL (high‐grade squamous intraepithelial lesion) were excluded from this analysis.

When cervical scrapes from the replication cohort were analyzed, promoter methylation levels remained statistically different between HSIL+ and Normal & LSIL groups (*p* = 0.0008 and *p *< 0.0001 for *hsa‐miR124‐2^me^* and *MAL^me^*, respectively). Moreover, accuracy and NPV were above 83% for *hsa‐miR124‐2^me^* and *MAL^me^*, but again with limited sensitivity (Table 1B). Then, HPV genotyping information was added to the methylation markers based on previous observations [6, 7]. Using this strategy, methylation markers achieved a sensitivity of nearly 70%, with specificity and accuracy above 75% (Table 1B). In comparison, the currently used strategy (HPV16/18 status + cytology) disclosed 65.7% (CI 95%: 62.1–69.3) specificity, with similar accuracy [67.2% (CI 95%: 63.9–70.6)] and higher sensitivity [78.3% (CI 95%: 69.8–86.7)].

Subsequently, we assessed the percentage of women sent to colposcopy and the false positive rate (FPR) for the current strategy and the new strategies proposed herein. Combined methylation + HrHPV genotyping resulted in a lower FPR and percentage of women sent to colposcopy (almost a one‐third reduction), maintaining higher accuracy (Table 1B). Importantly, when the analysis was restricted to cervical intraepithelial neoplasia (CIN) 3 or worse (CIN3+), the superiority of methylation allied to HPV genotyping was retained (Table 1C).

These results demonstrated the potential of methylation markers for triaging HrHPV‐positive women. Indeed, methylation markers have emerged as an alternative tool for triaging HrHPV‐positive women, referred to as colposcopy [3], including in the Portuguese population [5]. We selected the most promising genes based on that study and managed to optimize a ddMSP‐based assay for two of those genes (*hsa‐miR124‐2* and *MAL*). To the best of our knowledge, this is the first study using ddMSP to assess methylation status in cervical scrapes. This technology has been proven to avoid preamplification in low‐input samples and to better replicate tumor burden compared to RT‐qPCR [8].

Despite good accuracy, specificity, and NPV, methylation markers alone disclosed limited sensitivity. Nonetheless, the relatively large storage time of low‐concentrated samples allied to bisulfite treatment, might have negatively influenced our results. Remarkably, when methylation biomarkers were combined with HPV genotyping, performance increased, reaching nearly 70% sensitivity, and 80% specificity and accuracy. Remarkably, the performance of this algorithm emulates or even surpasses that of most host‐gene methylation‐based markers reported in the literature for HSIL+ detection in HrHPV‐positive women [3]. Interestingly, methylation markers have been previously combined with partial HrHPV genotyping [9], with a double negative result associated with a reduction in post‐test probability for CIN3+ lesions [9].

Furthermore, methylation markers are a promising tool to triage HrHPV‐positive women in self‐collected samples [3, 4], a methodology that has been increasingly adopted by screening programs. Likewise, Verhoef et al. reported on the potential of *MAL* and *miR124‐2* methylation for the triage of HrHPV‐positive women in self‐collected samples [10]. When several specificity cut‐offs were tested, threshold‐80 (standing for 80% specificity for CIN3+ detection) enabled an increase to 83% specificity for CIN2 or worse (CIN2+) detection, with a concomitant decrease in sensitivity to 33.8% [10]. When combined with HPV16/18 genotyping, the authors reported 55% specificity and 67.1% sensitivity [10]. In our study, 90% specificity for HSIL+ detection was set for defining the methylation cut‐off, negatively impacting sensitivity. However, adding HPV16/33 genotyping to the triage algorithm increased the sensitivity to values near those reported in the literature but with higher specificity [3, 10].

In conclusion, our findings disclose a novel strategy to improve triage for colposcopy of HrHPV‐positive women. Although future validations using prospective samples and management of HrHPV‐positive and triage‐negative women are still required, this strategy has the potential to increase the cost‐effectiveness of our regional screening program, providing a faster and more reliable referral pathway to the women enrolled.

## Author Contributions

S.S.: conceptualization, formal analysis, investigation, methodology, writing – original draft preparation, writing – review and editing. J.P.S.: formal analysis, investigation, methodology, writing – review and editing. B.A.M.: investigation. J.L.: investigation, writing – review and editing. V.T.: methodology, writing – review and editing. p.m.: resources, supervision, writing – review and editing. I.B.: resources, supervision, writing – review and editing. F.T.: resources, supervision, writing – review and editing. R.H.: conceptualization, funding acquisition, project administration, supervision, writing – review and editing. C.J.: conceptualization, funding acquisition, project administration, supervision writing – review and editing. All authors have read and approved the final manuscript.

## Ethics Statement

The study protocol was approved by the institutional review board of the Portuguese Oncology Institute of Porto (Comissão de Ética para a Saúde—CES‐371/2017). All the samples derived from routinely archived material were used after anonymization. Thus, according to Portuguese law, consent to use the samples was waived.

## Conflicts of Interest

The authors declare no conflicts of interest.

## Supporting information



Supporting Information

## Data Availability

Data are available from the corresponding author upon reasonable request.
