# Supplementary material for: Methylation‐Specific Droplet Digital PCR: Testing a Novel Triage Tool for HrHPV‐Positive Women in the Cervical Cancer Screening Program of Northern Portugal
Source: MedComm (2020). 2025 Jun 15;6(7):e70203. doi: 10.1002/mco2.70203 (PMC12167703; doi:10.1002/mco2.70203)
Supplement: Supplementary file 1 — Supporting Information [file MCO2-6-e70203-s001.docx]

**Supplementary Material – Salta et al. *Methylation-specific droplet digital PCR: testing a novel triage tool for HrHPV-positive women in the Cervical Cancer Screening Program of Northern Portugal***

**Supplementary Methods**

Study Population and Sample Selection

This retrospective study was carried out with the leftover samples primarily used for diagnosis. Thus, cervical scrapes from women enrolled in the Regional Cervical Cancer Screening Program of Northern Portugal were selected. All samples were collected in a methanol-based, buffered preservative solution (ThinPrep^TM^ PreservCyt^TM^), received at the Department of Pathology of Portuguese Oncology Institute of Porto (IPO Porto) and tested for HrHPV, as previously described ^26^. Briefly, 14 HrHPV genotypes (HPV-16, HPV-18, HPV-31, HPV-33, HPV-35, HPV-39, HPV-45, HPV-51, HPV-52, HPV-56, HPV-58, HPV-59, HPV-66, and HPV-68) were simultaneously detected using Anyplex^TM^ II HPV HR Detection kit (Seegene, Seoul, South Korea). For all samples, DNA was extracted and stored. In all HrHPV-positive cases, cytological examination was carried out by experienced cytopathologists. According to the current screening program flowchart, women who tested positive for non-HPV16/18 HrHPV and have a cytological result of Atypical Squamous Cell of Undetermined Significance (ASC-US) or worse are referred to colposcopy; women positive for HPV16 or HPV18 are directly referred to colposcopy, regardless of cytological result; women positive for non-HPV16/18 HrHPV and a cytological result of Negative for Intraepithelial Lesion or Malignancy (NILM) repeat screening after one year. All HrHPV-negative cases are invited to repeat the cervical cancer screening after five years.

For this study, two patient cohorts were selected: exploratory and replication cohorts. Relevant clinical data is depicted in Supplementary Tables S1 and S2.

For the exploratory series, 62 samples were selected from a patient cohort previously characterized ^18^ who tested positive for HrHPV and were referred to colposcopy between March and May of 2019. Only patients who had colposcopy information available were selected. Patients who did not attend colposcopy were excluded from this analysis.

For the replication series, sample selection was performed as depicted in Figure 1. All samples received at the Department of Pathology of IPO Porto in January 2020 which tested positive for HrHPV were considered eligible, except those without clinical information or enough DNA stored, which were excluded from analysis. This study was approved by the institutional review board of IPO Porto (Comissão de Ética para a Saúde – CES-371/2017). All samples derived from routinely archived material, and were used after anonymization. Thus, according to Portuguese law, consent to use the samples was waived.

Methylation analyses

From all cervical scrapes (exploratory and replication series), only DNA leftovers from the regional screening program (extracted in an automated system as previously described ^26^) were used. DNA was stored at -80ºC until further use.

Bisulfite treatment was performed for all samples using the EZ DNA Methylation-Gold Kit (Zymo Research, Orange, California), as previously described ^18^. For control purposes, Human HCT116 DKO Methylated DNA [positive control (Zymo Research, Orange, California)] and Human HCT116 DKO Non-Methylated DNA [negative control (Zymo Research, Orange, California)] were quantified using Qubit dsDNA BR Assay Kit (Thermo Fisher Scientific, Waltham, Massachusetts), bisulfite-treated, and eluted to the final concentration of 2.5ng/µL.

Further, ddMSP reactions were prepared with 11µL ddPCR Supermix for Probes (No dUTP) (Bio-Rad, Hercules, California), 2µL of bisulfite-treated DNA, primers [400nM], and probes [250nM for *hsa-miR124-2* and *MAL* and 600nM for *Albumin*] and bidistilled water up to final volume 22µL. Droplets were generated using Droplet Generator QX200 (Bio-Rad, Hercules, California). A positive, a negative, and a non-template control were included in each plate. The PCR run was set as follows: 95ºC for 10 minutes, 45 cycles of 94ºC for 15 seconds and 57.3ºC for 1 minute, 98ºC for 10 minutes. All the steps were run with a 2.5ºC ramp/rate in C1000 Touch (Bio-Rad, Hercules, California). After PCR, the plate was read on the QX200 Droplet Reader (Bio-Rad, Hercules, California). Plates were analyzed using QX Manager 1.2 Standard Edition (Bio-Rad, Hercules, California). Wells were considered valid if at least 10,000 droplets were read.

The Limit of Blank (LOB) and the Limit of Detection (LOD) were calculated for each target as previously described ^27^. Briefly, 30 wells with negative control were run. The LOB was set at one droplet and two droplets for *hsa-miR124-2* and *MAL* methylation, respectively*.* The LOD was five and six droplets for *hsa-miR124-2* and *MAL* methylation, respectively. Methylation ratios for each target gene were calculated by dividing copies/20µL of the target by copies/20µL of albumin. Samples were considered valid for analysis if albumin concentration was equal to or higher than 300 copies/20µL (15 copies/µL).

Statistical Analysis

Two-group comparisons were performed using the Mann-Whitney U test. To assess the biomarker performance of the methylation markers for HSIL+ detection (according to histological results), Receiver Operator Characteristic (ROC) curves were constructed, and the area under the curve (AUC) was calculated. Normal (including normal cervix, metaplasia, and cervicitis) and Low-grade squamous intraepithelial lesions (LSIL) samples were considered the control group. The cut-off for each target gene was established at 90% specificity based on the ROC curve in the exploratory series to categorize the samples as methylated or unmethylated. the cut-off was set at 0.01142 for *hsa-miR124-2^me^* and at 0.00034 for *MAL^me^*. The cut-off established in the exploratory series was applied directly in the replication series. When panels were used, samples were considered positive if at least one parameter was positive.

Furthermore, sensitivity, specificity, PPV, NPV, accuracy, and corresponding 95% confidence intervals (CI) for HSIL+ detection were calculated. The referral rate was calculated as the percentage of women who tested positive for the biomarkers among all women tested. Pre- and Post-test Probabilities were calculated as previously described ^28,29^. Two-tailed p-values were derived from statistical tests using IBM SPSS Statistics (Version 27.0, Chicago, Illinois), and a p-value <0.05 was considered statistically significant. Graphs were assembled using GraphPad 8 Prism (GraphPad Software, Boston, Massachusetts).

**Supplementary Table S1 –** Clinicopathological features of women cohorts.

| **Clinical features** | Exploratory cohort n=62 | Validation cohort n=750 |
| --- | --- | --- |
| Age [median (range)] | 41 (24-61) | 39 (25-64) |
| HPV result [n, (%)]^1^  HPV-16  HPV-18  HPV-31  HPV-33  HPV-35  HPV-39  HPV-45  HPV-51  HPV-52  HPV-56  HPV-58  HPV-59  HPV-66  HPV-68  Multiple infections with HPV-16/18  Multiple infections without HPV-16/18 | 9 (14.5%)  2 (3.2%)  4 (6.5%)  2 (3.2%)  1 (1.6%)  1 (1.6%)  1 (1.6%)  1 (1.6%)  2 (3.2%)  3 (4.8%)  2 (3.2%)  1 (1.6%)  4 (6.5%)  3 (4.8%)  9 (14.5%)  17 (27.4%) | 53 (7.1%)  10 (1.3%)  66 (8.8%)  18 (2.4%)  19 (2.5%)  40 (5.3%)  16 (2.1%)  50 (6.7%)  58 (7.7%)  44 (5.9%)  31 (4.1%)  22 (2.9%)  49 (6.5%)  63 (8.4%)  54 (7.2%)  157 (20.9%) |
| Cytological result [n*,* (%)]  NILM  ASC-US  LSIL  ASC-H  HSIL  AGC  Insufficient/unsatisfactory | 6 (9.7%)  32 (51.6%)  12 (19.4%)  7 (11.3%)  3 (4.8%)  1 (1.6%)  1 (1.6%) | 513 (68.4%)  138 (18.4%)  53 (7.1%)  30 (4.0%)  12 (1.6%)  0 (0%)  4 (0.5%) |
| Histological result [n*,* (%)]  No lesion^2^  LSIL  HSIL  SCC/AIS  Not available/ not applicable | 16 (24.2%)  14 (22.6%)  17 (27.4%)  0 (0%)  15 (24.2%) | 85 (11.3%)  179 (23.9%)  90 (12.0%)  2 (0.3%)  394(52.5%) |
| ^1^ – The sum of each hrHPV genotype cases percentage is higher than 100% due to the existence of cases with multiple HrHPV infections; ^2^ In this group are included cervicitis, metaplasia, and the normal histology. **Abbreviations:** AGC – Atypical glandular cells; AIS – Adenocarcinoma *in situ*; ASC-H – Atypical squamous cells - cannot exclude high-grade squamous intraepithelial lesion; ASC-US – Atypical squamous cells of undetermined significance; HSIL – High-grade squamous intraepithelial lesions; LSIL – Low-grade squamous intraepithelial lesions; n – number; NILM – Negative for intraepithelial lesion or malignancy; SSC – Squamous cell carcinoma. | | |
